# Evaluation of Shear Strength and Stiffness of a Loess–Sand Mixture in Triaxial and Unconfined Compression Tests

**DOI:** 10.3390/ma17153831

**Published:** 2024-08-02

**Authors:** Matylda Tankiewicz, Magdalena Kowalska, Jakub Mońka

**Affiliations:** 1 Department of Civil Engineering, Wrocław University of Environmental and Life Sciences, Norwida 25, 50-375 Wrocław, Poland; 2 Department of Geotechnics and Roads, Silesian University of Technology, Akademicka 5, 44-100 Gliwice, Poland; magdalena.kowalska@polsl.pl

**Keywords:** loess, triaxial test, piezoceramic elements, unconfined compression, stiffness, shear strength

## Abstract

Mechanical soil parameters are not constants and can be defined in various ways. Therefore, determination of their values for engineering practice is difficult. This problem is discussed based on results of piezoceramic element tests and triaxial tests (unconfined and confined) on loess specimens improved by compaction and sand admixture (20% by weight). The study indicated also the effectiveness of this simple method of loess stabilization. The influence of specimen size, draining conditions, stress and strain state, and different calculation methods on the evaluation of basic mechanical parameters were analyzed. The initial shear and Young’s moduli, the degradation of secant moduli with strain, tangent moduli, and Poisson’ ratio were determined. The results showed that the shear strength parameters are much less sensitive to the test variables than the stiffness parameters are. In triaxial tests, the strength criterion adopted, the sample size, and the drainage conditions influenced the measured value of cohesion, with a much smaller impact on the angle of internal friction. On the other hand, the adopted definition of the parameter and the range of strains had the greatest influence on the value of the stiffness modulus. Moreover, larger specimens were usually found to be stiffer.

## 1. Introduction

Evaluation of soil shear strength and stiffness is one of the main tasks of geotechnical investigation. These properties are crucial at every stage of the ‘lives’ of geoengineering structures for their safe and economical design, execution, monitoring, improvement, or demolition. Due to the complicated nature of soils, the mechanical parameters cannot be treated as constants [1], as they depend on several factors, such as current stress and strain conditions, the genesis of the deposit, mineralogical composition, loading history and loading velocity, saturation degree, etc. Even if the study is limited to the results of laboratory tests, representing just one point of the subsoil, the data can be interpreted in various ways, and, thus, give different end results. As there are no well-defined common guidelines for the selection of the particular test methods and calculation procedures, this may be very confusing for civil engineers.

The required set of parameters needed to describe the complete soil behavior depends on the constitutive model that is to be used in the design [2]. However, the basic set, found in practically every technical document and used in most models, includes the following parameters: cohesion *c* and angle of internal friction *φ* to define the shear strength boundary, and shear modulus *G* or Young’s modulus *E*, and Poisson’s ratio *ν*, for soil stiffness in the pre-failure stage. They all can be evaluated in basic field or laboratory tests, although the definitions of these parameters and the conditions in which they should be determined are not always clear.

In this paper, the parameters of shear strength and stiffness, i.e., *c’*, *φ’*, *E*, *G,* and *ν*, were evaluated, based on results of conventional triaxial and unconfined compression experiments, supplemented by piezoceramic element tests. The study was carried out on a saturated, remolded, and compacted loess with 20% sand admixture (by weight). This particular soil was chosen because the pre-failure behavior and shear strength of this material have not been well recognized yet. Compared to the research works on undisturbed loess [3,4,5,6,7,8,9], there are much fewer studies referring to the mechanical parameters of the remolded and compacted loess [7,10,11,12,13,14,15]. Several studies focused on chemical stabilization, e.g., [16,17,18,19], but very few papers [20,21] refer to loess improved by the addition of a coarser fraction (e.g., sand), which is one of the simplest, yet poorly recognized, solutions. Researchers usually concentrate on selected types of behavior and parameters. For instance, (a) Zhang et al. [19], Ma and Ma [22], and Li et al. [23] investigated the unconfined compression strength of compacted loess; (b) Kim and Kang [11] and Zhang et al. [19] obtained direct shear strength parameters; (c) Zhong and Liu [9], as well as Li et al., [23] tested undisturbed loess in triaxial apparatus to determine its behavior and shear strength; (d) Ma et al. [12] and Xu et al. [15] assessed *c’* and *φ’* in triaxial tests on consolidated specimens that were sheared in undrained conditions; (e) Rinaldi et al. [5,6] and Wang et al. [8,13] investigated the small-strain shear modulus *G*_0_ under isotropic consolidation, using bender elements; (f) Liu et al. [20] determined the effect of water and sand content on small-strain stiffness, using a resonant column; (g) Song et al. [7] examined the *G*_0_ and damping of undisturbed loess in torsional resonant column tests; (h) Bowers [24] evaluated Poisson’s ratio and the initial tangent Young’s modulus for unsaturated loess. The literature results point out the influence of grading, water content, and void ratio on the material behavior. Contrary to the mentioned analyses, in this study, special attention was paid to the various factors influencing the soil strength and stiffness, such as saturation (low or complete), confining stress (50–400 kPa), strain (ca. 0.01–10%), draining conditions, specimen size, and strain measurement method. This allowed a comprehensive comparison of the mechanical parameters evaluated based on different test procedures and interpretation techniques. Such an approach is rare, yet it is necessary for better understanding of material behavior [25]. In addition, the majority of studies refer to the loess from the Chinese Loess Plateau [7,8,9,12,13,14,15,19,20,22,23]; only few consider loess from America [5,6,11,24], and much less is known about the European loess. Therefore, the results obtained in this research for loess deposited in Poland are filling a knowledge gap. Some preliminary outcomes referring to the stiffness of the loess–sand mixture have been presented in [21]. This study is an extension and continuation of that work. The results are also compared with those published for similar soils.

## 2. Mechanical Parameters Interpretation

Cohesion and the angle of internal friction are the parameters (initial value and slope) of a linear Coulomb function, approximating the failure boundary in the shear stress–normal stress (*τ*–*σ*‘) space. The boundary is generally curvilinear [26], thus the linear approximation is valid only for a given range of *σ*. The failure boundary can be defined in various ways—it can describe, e.g., the peak shear strength, as well as critical (steady), unstable, or residual states, giving different values of the parameters. This also means that the cohesion and angle of internal friction should not be treated as intrinsic soil properties. It is worth mentioning that *c* = 0 kPa, according to the critical state theory, which is also consistent with the approach presented by Santamarina [27] and Schofield [28].

Although commonly used in all design situations, Young’s modulus and Poisson’s ratio are, theoretically, relevant only there, where the minor *σ*_3_ and intermediate *σ*_2_ principal stress tensor components are zero. This can be simulated in unconfined compression tests. The parameters are calculated with the following Equations (1) and (2):

(1) $$E=\frac{∆{\sigma ’}_{1}}{{∆\varepsilon }_{1}}$$

(2) $$\nu =\frac{∆\varepsilon_{3}}{{∆\varepsilon }_{1}}$$

where Δ*σ’*_1_ and Δ*ε*_1_ are, respectively, the increments of axial (vertical, major) effective stress and strain, and Δ*ε*_3_ is the increment of strain in the direction perpendicular to the loading (horizontal, minor). Unconfined compression rarely occurs in a real subsoil subjected to loading and is simply infeasible for some soils (e.g., loose sands). Therefore, in geotechnics, triaxial tests on confined specimens are much more preferred. In triaxial compression, the horizontal components of the strain and stress tensor are equal, so, based on the theory of elasticity [29], the Poisson’s ratio and the Young’s modulus during shearing, at Δ*σ’*_1_ > Δ*σ’*_3_, shall be estimated with the following Equations (3) and (4):

(3) $$\nu =\frac{{∆\varepsilon }_{1}\cdot ∆{\sigma^{’}}_{3}-{∆\varepsilon }_{3}\cdot ∆{\sigma^{’}}_{1}}{∆{\sigma ’}_{3}\cdot \left({∆\varepsilon }_{1}-2{∆\varepsilon }_{3}\right)+∆{\sigma ’}_{1}\cdot ∆\varepsilon_{3}}$$

(4) $$E=\frac{\left(∆{\sigma^{’}}_{1}+2∆{\sigma^{’}}_{3})(∆{\sigma^{’}}_{1}-∆{\sigma^{’}}_{3}\right)}{∆{\sigma ’}_{3}\cdot \left({∆\varepsilon }_{1}-2{∆\varepsilon }_{3}\right)+∆{\sigma ’}_{1}\cdot ∆\varepsilon_{3}}=\frac{∆{\sigma^{’}}_{1}-2\nu \cdot ∆{\sigma^{’}}_{3}}{∆\varepsilon_{1}}$$

where Δ*ε*_1_, Δ*ε*_2_, Δ*ε*_3_ and Δ*σ’*_1_, Δ*σ’*_2_, Δ*σ’*_3_ are the major, intermediate, and minor principal strains and stresses, respectively. During an undrained shearing, the Equation (4) reduces to Equation (1), because the Poisson’s ratio *ν* can be assumed to be equal to 0.5, while in a drained test, as Δ*σ’*_3_ = 0, Equation (4) reduces to the following Equation (5):

(5) $$E=\frac{∆q}{{∆\varepsilon }_{1}}$$

where Δ*q* is the increment of effective or total stress intensity, being the difference between the major and minor principal stress (*q* = *q*’ = *σ’*_1_ *− σ’*_3_). The *E* values depend on the drainage conditions, so two types of elastic moduli are usually distinguished—‘drained’ *E’* and ‘undrained’ *E*_u_.

The shear modulus *G* is theoretically defined as the ratio of shear stress *τ* and shear strain *γ* in the conditions of simple shearing, i.e., with zero normal stress tensor components (mean effective stress *p’* = 0) and with no volume change (*ε*_vol_ = 0). It is considered independent of drainage conditions [30]. Although the true simple shear can be simulated only in a simple shear apparatus [31], *G* is often evaluated in triaxial compression tests, using the following Equation (6):

(6) $$G=\frac{∆\tau }{∆\gamma }=\frac{0.5(∆{\sigma^{’}}_{1}-∆{\sigma^{’}}_{3})}{{∆\varepsilon }_{1}-{∆\varepsilon }_{3}}=\frac{∆q}{3{∆\varepsilon }_{s}}$$

where *ε*_s_ is the strain intensity calculated as 2/3 of the difference between the major (vertical) *ε*_1_ and minor (horizontal) *ε*_3_ principal strains. The influence of volumetric strain can be treated as negligible in undrained conditions. In such a case, *ε*_s_ corresponds to *ε*_1_ and *G* to 1/3 of the elastic ‘undrained’ modulus *E*_u_. However, in a standard test with constant confining effective stress, this approach does not meet the remaining simple shear condition of Δ*p’* = 0.

Both the shear and elastic moduli can be calculated as the secant or tangent (c.f. Figure 1 in [21]). They strongly depend on the current values of stress and strain, and on the previous loading history. In triaxial compression, the secant modulus is always positive, while the tangent modulus decreases to zero at the maximum stress intensity *q*_f_ and becomes negative if the soil softens with strain. Constitutive modelling often uses a specific value of the secant modulus obtained in a monotonic loading at 50% of *q*_f_, named *E*_50_. 

It is well known that the soil stiffness decreases with strain [32,33] and that various strain measurement techniques have to be used to accurately describe the degradation in triaxial tests [21,25,34]. A complete set includes external and local (on-sample) strain transducers to read large (>10^−3^) and small (10^−6^–10^−2^) strains, respectively, and a pair of piezoceramic elements (bender–extenders) for the very small strain range (<10^−5^). Piezoceramic elements (PE) allow non-destructive measurement of the velocity of shear wave (S-wave) and compression wave (P-wave), *V_S_* and *V_P_*, passing through a soil specimen. If the current bulk density of the soil *ρ* is known, the initial (maximum) shear modulus *G*_0_ can be calculated based on the following Equation (7):

(7) $$G_{0}=\rho \cdot V_{S}^{2}$$

It is believed that PE generate strains smaller than 0.001% [35] and that in this range soil behaves as a linearly elastic material. Based on the theory of elasticity and assuming that the soil is isotropic, the initial Young’s modulus *E*_0_ can be calculated from the following Equation (8):

(8) $$E_{0}=2G_{0}(1+\nu_{0})$$

There is no distinction between the secant and tangent initial moduli. The initial Poisson’s ratio *ν*_0_ can be determined from the PE tests [36] based on the following Equation (9):

(9) $$v_{0}=\frac{0.5{\left(\frac{V_{P}}{V_{S}}\right)}^{2}-1}{{\left(\frac{V_{P}}{V_{S}}\right)}^{2}-1}$$

It shall be noted that the P-wave velocity depends strongly on the degree of saturation— therefore, this method of evaluation of the Poisson’s ratio is not recommended for saturated soils.

## 3. Materials and Sample Preparation

**Figure 1:**
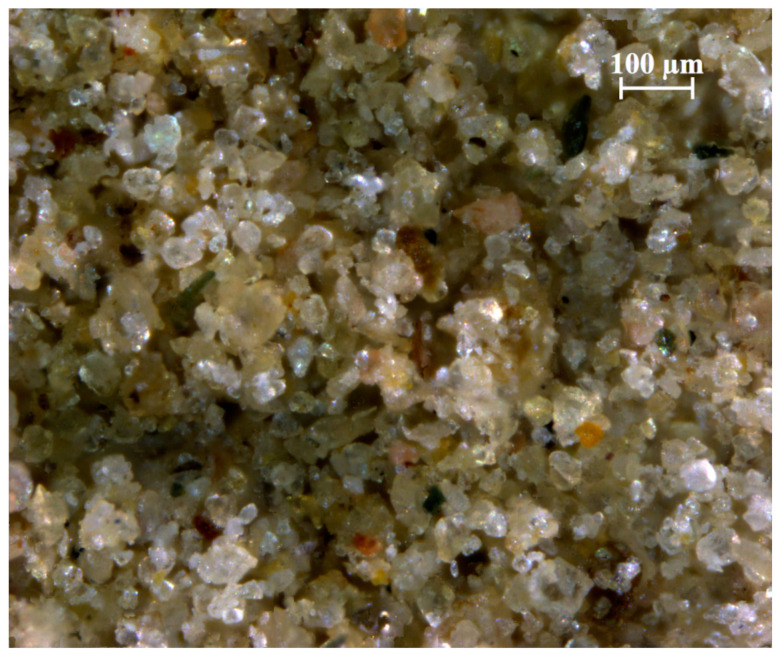
Microscopic image of the tested loess (ZEISS SteREO Discovery.V20 stereomicroscope, ZEISS, Oberkochen, Germany).

The study was carried out on a loess from the Sudety forelands deposit in Trzebnica Hills, in the neighborhood of the city of Wrocław (Poland). The site belongs to the northern European loess belt [37]. Studies on its sedimentology, paleopedology, and stratigraphy can be found in [38,39,40]. In this area, the loess deposits are typically 4–6 m thick and contain large amounts of sand and clay fractions, with organic matter and calcium carbonate contents of approximately 1% and 0–5%, respectively. The soil sample was taken directly from a slope exposure. From the microscopic image shown in Figure 1, it is clear that the silty particles are typical for loess—they are mostly isometric and consist of quartz crystal fragments. 

**Figure 2:**
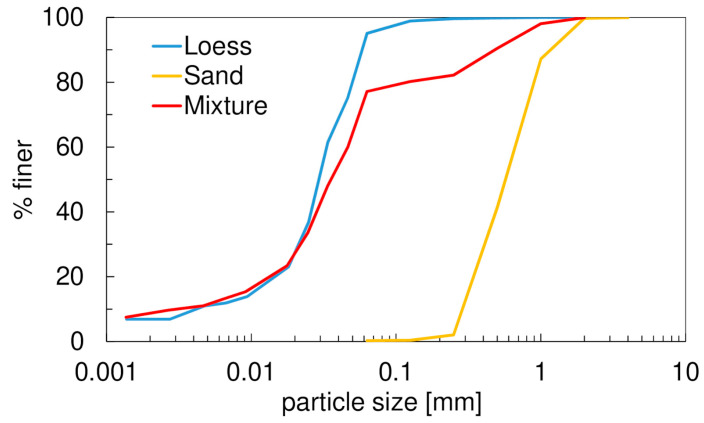
Grain size distribution of loess, sand, and loess–sand mixture.

The material was passed through a 1 mm sieve, dried, and mixed with a medium quartz sand in the proportion of 80%:20% by weight. The grain size distribution curves of the sand, loess, and the sand–loess mixture (after the removal of calcium carbonate; ISO 17892-4 [41]) are shown in Figure 2. The physical parameters of the mixture, namely sand (Sa), silt (Si), and clay (Cl) contents, mean grain size *d*_50_, coefficients of uniformity *C_U_* and curvature *C_C_*, plastic limit *w_P_*, liquid limit *w_L_* [42], plasticity index *I_P_ = w_L_ − w_P_*, colloidal activity *A*, loss on ignition *LOI* at 600 °C [43], particle density *ρ_s_* [44], optimum moisture content *w_opt_*, and maximum dry density *ρ_d_._max_* [45], are shown in Table 1. The compaction parameters were established using standard Proctor energy of 0.6 J/cm^3^. To prepare the material for further tests, an amount of water corresponding to *w_opt_* was admixed to the dry loess–sand mixture and left for an overnight rest in an airtight container. Two specimen sizes were used: 70 mm/140 mm and 38 mm/76 mm (diameter/height). Due to equipment limitations, slightly different procedures were applied to prepare specimens. The larger specimens were compacted in 3 layers with the standard Proctor energy directly in a split mold of appropriate dimensions. The smaller ones were cut out from the larger specimens by means of a cutting ring. Despite the different preparation procedures, the bulk densities of the specimens, irrespective of the size, differed by not more than 1%. 

## 4. Methodology

The prepared loess–sand specimens were subjected to unconfined compression tests, conventional triaxial compression tests, and bender–extender element tests in order to determine the shear strength and stiffness parameters of the material under various stress–strain and drainage conditions. A summary of all specimens tested is shown in Table 2 and Table 3 with the initial values of the bulk density *ρ*_0_ and the void ratio *e*_0_. The following codes are used for the particular specimens: type of test/diameter for unconfined compression tests, e.g., UC/70, and type of test/diameter/confining stress for triaxial compression tests, e.g., CIU/38/100, CID/70/200. The triaxial tests conducted with the vertical stress paths are indicated with a letter V at the end, e.g., CID/70/200/V.

The unconfined compression tests were carried out, according to ISO 17892-7 [46] on six UC/38 and six UC/70 specimens. The specimens were unsaturated, with the average degree of saturation equal to 69%. All were compressed with an axial strain velocity of 25%/hour until a considerable drop in the vertical stress was observed. This velocity allowed for more than 10 readings to be taken before failure and provided the required testing time of between 2 and 15 min. The height change in the specimens was measured with an external LDT sensor. Additionally, LVDT on-specimen transducers measured the vertical strain and the change in the specimen diameter. The unconfined compression strength, the average *E*_50_ modulus, and the Poisson’s ratio *ν* were determined.

The conventional triaxial compression tests were performed according to ISO 17892-9 [47]. The specimens were saturated at a back pressure of 450 kPa, achieving a Skempton’s *B* value above 0.95. They were isotropically consolidated at the selected effective confining pressure *σ*’_3_ (consolidation pressure *σ’_C_*) in the range of 50–400 kPa. Next, the specimens were sheared, maintaining the cell pressure constant. Two specimens were subjected to a vertical stress path (constant *p’* = 200 or 350 kPa). The vertical displacement rate of the load frame was equal to 0.64%/h and 2.50%/h under the drained conditions (CID) and undrained (CIU) conditions, respectively. The shearing stage was continued until at least 15% of axial strain was achieved or until a clear shear failure surface was identified. The vertical deformation of the specimens was measured using external vertical displacement transducers. Local LVDT or Hall-effect sensors (c.f. Table 3) were additionally used on selected specimens. Unless stated otherwise, the strain calculations were based on the readings of the local displacement sensors, within their range. For larger strains and in the tests where the local sensors were not available, the readings of the external vertical displacement sensor and the volume change measurements were applied. The PE element tests (PETs) were performed on saturated and consolidated specimens 70 mm in diameter that were later subjected to drained shearing. Details of the PET methodology applied in this study can be found in [21]. The readings were taken at *σ’_C_* (equal to the mean effective stress *p’*_0_) within the range of 10–400 kPa. The triaxial compression and PETs were used to estimate the soil shear strength, as well as the values of the tangent and secant *E* and *G* moduli and their degradation with strain in drained and undrained conditions. The Poisson’s ratio *ν* was also assessed. Various approaches were applied, as presented in Section 2. 

## 5. Results and Discussion

### 5.1. Strength and Stiffness in Unconfined Compression

The average values of unconfined compression strength (*UCS*), secant Young’s modulus (*E*_50_), and Poisson’s ratio (*ν*_50_) (at the same strain for which *E*_50_ was estimated) obtained in UC tests, together with the standard deviations (SD), are shown in Table 4. The values were calculated separately, based on the measurements from local (*loc*) and external (*ex*) displacement sensors. 

**Figure 3:**
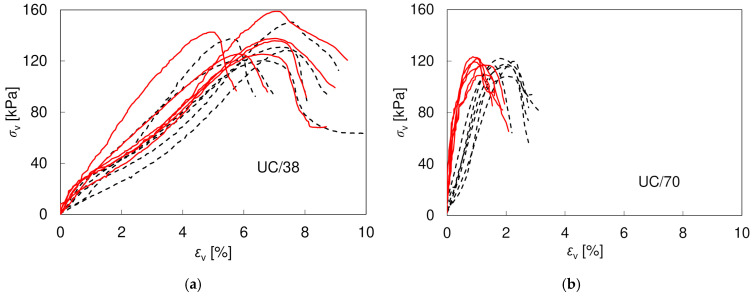
Shear characteristics for six UC/38 (**a**) and six UC/70 (**b**) specimens based on external sensors (black dashed lines) and local sensors (red continuous lines).

In Figure 3, showing the stress–strain curves for all the specimens tested in unconfined compression, quite a large scatter can be observed, which is common for this type of test [22]. It is visually larger in UC/38 specimens (applying the same scale) and may result from the fact that smaller specimens are naturally less uniform, which is confirmed by larger SD values for the density and void ratio in Table 2. The differences in *UCS* obtained using *loc* and *ex* measurements for one specimen size result from the different area measurements and are negligible (<5%). The *UCS* values obtained are smaller than those reported for compacted natural loess (0.26–2.1 MPa) by other researchers [19,22,23]. It shall be noted, however, that the mentioned results referred to the loess from Chinese Loess Plateau, which has a much higher natural calcium carbonate content, responsible for an increase in strength. In addition, the authors of [22] reported a large influence of moisture content on *UCS* values, and, therefore, the data cannot be directly compared. In this study, the effect of moisture content was not analyzed. However, the main factor responsible for the smaller *UCS* values is definitely the increased content of sand, resulting from the larger size of the voids and the smaller interparticle suction. The average *UCS* obtained for the UC/38 specimens is only 13% or 17% greater than for the UC/70 specimens, but it was achieved at much larger vertical strain (*ex*: 5.5–7.5% vs. 1.5–2.5% or *loc*: 5.0–7.5% vs. 1.0–1.1%), eventually giving much smaller Young’s modulus values. This may result from both different sample sizes and different preparation procedures and requires further investigation. The average *E*_50_ moduli of the UC/38 specimens seem to not depend much on the method of measurement (*loc* or *ex*), while in the UC/70 specimens the stiffness measured with the external LDT sensor is much smaller than the one based on the on-specimen transducers. As the tests of different specimen sizes were performed also in different apparatuses, this may be connected with the different sensitivity of the sensors used or the typical problems encountered in conventional deformation measurements, such as seating or bedding errors [48,49].

**Figure 4:**
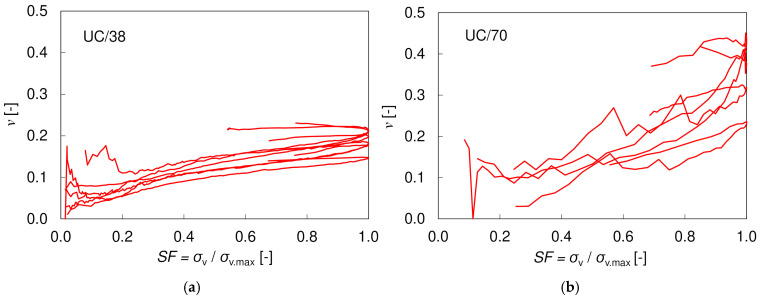
Poisson’s ratio values for (**a**) UC/38 and (**b**) UC/70 specimens based on local sensors.

The Poisson’s ratio *ν* was calculated based on the local strain measurements. Figure 4 presents the variation of *ν* with stress ratio *SF* = *σ_v_*/*σ_v.max_*. The results for UC/70 specimens showed a large scatter; thus, only the four most representative specimens have been shown. Regardless of the size of the specimen, the Poisson’s ratio increases gradually with SF. A similar phenomenon was reported by Bowers [24] in triaxial tests on unsaturated loess. At *SF* = 0.5 (denoted as *ν*_50_), the Poisson’s ratio is equal to approximately 0.14 (for both sizes), which is consistent with the results presented in [50] and close to 0.1, which is a value typically assumed for unsaturated soils [51].

### 5.2. Shear Strength in Triaxial Compression

**Figure 5:**
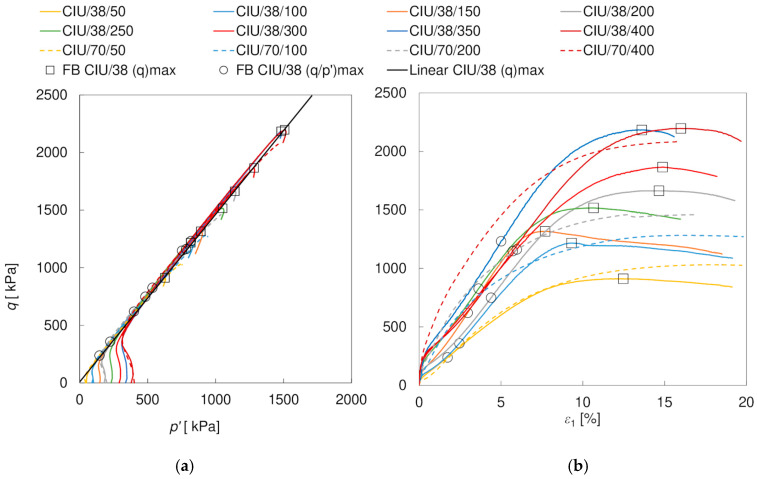
Stress paths (**a**) and shear characteristics (**b**) for CIU/38 and CIU/70 specimens.

**Figure 6:**
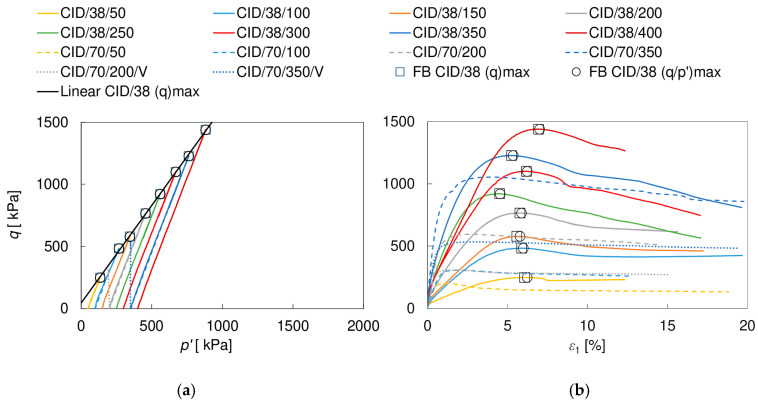
Stress paths (**a**) and shear characteristics (**b**) for CID/38 and CID/70 specimens.

The stress paths *p’*–*q* and the shearing characteristics *ε*_1_–*q* obtained in the triaxial compression tests are shown in Figure 5 and Figure 6. The circular and square markers indicate the points representing the criteria (*q/p’)*_max_ and (*q*)_max_, respectively. For clarity, they were presented only for CIU/38 and CID/38 specimens. It is clear that the behavior of the soil depends strongly on the drainage conditions, which is mainly due to the different shapes of the stress paths. In the drained tests, the peak shear strength (*q*)_max_ is much smaller than in the undrained tests. It is also achieved at smaller axial strains: about 1.1–4.0% and 4.5–6.9% in the CID/70 and CID/38 specimens, compared to >15% and 7.7–16.0% in the CIU/70 and CIU/38 specimens, respectively. For drained tests, the peak corresponds to the failure condition (*q*/*p’)*_max_, while in undrained tests, the (*q*/*p’)*_max_ is achieved at significantly lower strains—approximately 0.8–1.5% and 1.7–5.7% in the CIU/70 and CIU/38 tests, respectively. The strain at which the failure boundary is reached is, therefore, affected not only by the type of drainage but also by the size of the specimen. Furthermore, the stress–strain curves show more distinct peaks in drained conditions and in the smaller specimens. The influence of the size of the specimen on the softening rate was also observed by Hu et al. [52] and may be related to the fact that in the larger specimens more than one distinct failure surface was observed, causing a different stress redistribution. The size of the specimen also played a role in the evolution of excess pore pressure *u* in the undrained tests, contrary to the observations of Omar and Sadrekarimi [53] in their tests on sand. For both specimen sizes, the excess pore pressure initially increased with axial strain, after which it started to decrease, reaching negative values (suction). For large specimens, the maximum *u* values were obtained at strains of approximately 0.4–0.8%, that is, before (*q*/*p’)*_max_ was reached, and negative *u* values were obtained at strains of approximately 1.2–3.8%, that is, after (*q*/*p’)*_max_ and long before *(q)_max_* were achieved. For small specimens, usually both the maximum and negative values appeared before reaching the (*q/p’)_max_*, that is, at about 0.3–1.5% and 1.3–6.2%, respectively. For drained tests, after an initial decrease in volume with the peak at approximately 0.2–1.6% (CID/70) or 2.0–3.5% (CID/38) of axial strains, the attainment of negative volumetric strain values occurred approximately when the failure condition was reached (*(q)_max_* ~ (*q/p’)_max_*), irrespective of the specimen size. It is worth mentioning that the strain evolution of the CID/70/350/V and CIU/70/400 specimens, as well as the CID/70/200/V and CIU/70/200 specimens, with very similar stress paths, was practically the same until the stress path reached the failure boundary; then, under drained conditions, the specimens began to yield, while in the undrained tests, as the stress path ‘slid’ along the failure boundary, a constant increase in shear strength was observed. This strain hardening phenomenon is typical for strongly dilatant well-compacted loess, cf. e.g., [12,15].

The effective strength parameters (cohesion *c’* and angle of internal friction *φ’*) of the Mohr–Coulomb failure boundary (FB) are summarized in Table 5. They were calculated based on two selected failure criteria: (*q*/*p’)*_max_ and (*q*)_max_. The steady or critical state, in which the mass is continuously deforming at constant volume, constant normal effective stress, constant shear stress, and constant velocity [54], was not observed until 15% axial strain; therefore, this criterion was not considered. It should be noted that within the stress range analyzed, the FBs considered were rectilinear, with a coefficient of determination of R^2^ ≥ 0.999. The selection of the failure criterion only had an influence on the results of the CIU tests, as, in the drained tests, both criteria occurred at almost the same strains. With the (*q*)_max_ considered as the shear strength, slightly smaller *φ’* values were obtained at (forced) zero cohesion. As only very slight strength degradation and pore pressure change were observed in these tests after (*q*)_max_ was reached, these results are considered to be close to the steady or critical state. There is no clear dependency between the angle of internal friction and the size of the specimen, but it appears that, except for the undrained test at the (*q*/*p’*)_max_ criterion, *φ’* becomes slightly smaller (by 1.4–2.4°) in the larger specimens, which is similar to the trends observed in sands [52,53]. Cohesion also tends to decrease in the larger specimens. The angle of internal friction values are high, when compared to the results obtained by other researchers for natural compacted loess, which have been summarized in Table 6. This is mainly due to the lower clay content and the higher sand content in the loess–sand mixture tested in this study, indicating the effectiveness of the improvement in terms of the angle of internal friction. The relatively lower cohesion can be explained by the smaller amount of clay fraction (and so clayey minerals), which is believed to coat the individual silt grains and to be the main cementing agent in the natural loess [55], next to calcite. It should be noted that the shear strength parameters obtained in this investigation correspond well to the values obtained by Xu et al. [15] for the loess with similar physical properties.

### 5.3. Stiffness in Triaxial Compression

#### 5.3.1. Initial Shear Modulus G_0_, Poisson’s Ratio ν_0_, and Young’s Modulus E_0_

**Figure 7:**
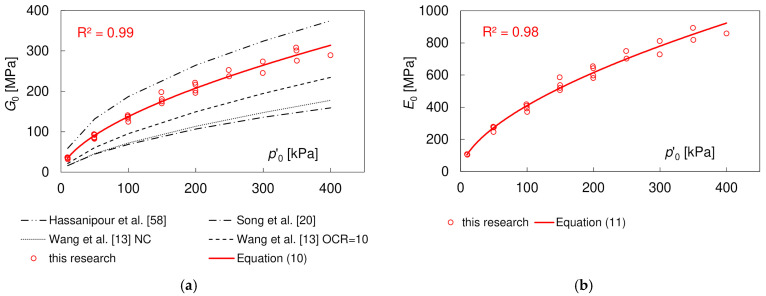
Influence of the mean effective stress *p’*_0_ on (**a**) the initial shear modulus *G*_0_ and (**b**) the initial Young’s modulus *E*_0_ [9].

The results of the initial shear moduli *G*_0_, determined in the PETs, in relation to the mean effective stress *p’*_0_, are given in Figure 7a (after [21]). The *G*_0_ values were compared with the results of three other studies on similar soils: (a) by Wang et al. [13] on normally consolidated and over-consolidated compacted loess (Sa = 5%, Si = 86%, Cl = 9%; initial bulk density *ρ*_0_ = 1.7 g/cm^3^), (b) by Song et al. [20] on undisturbed Lanzhou loess wetted to *w* ≈ 16% (Sa = 5%, Si = 85%, Cl = 10%, *ρ*_0_ = 1.45–1.75 g/cm^3^), and (c) an empirical equation proposed by Hassanipour et al. [58] for well-compacted sand–clay mixtures with Sa ≤ 60%. The *G*_0_ values of the loess–sand mixture in our research were almost twice as large as of the loess with no admixtures reported in the literature, indicating the strong positive influence of compaction and increased sand content. They were, however, lower than in the sand–clay mixture.

The dependence between the initial stiffness modulus of the loess–sand mixture and *p’*_0_ can be expressed with the use of a simple power law widely accepted in soil dynamics, resulting in the following Equation (10):

(10) $$\frac{G_{0}}{p_{a}}=1370\cdot {\left(\frac{{p’}_{0}}{p_{a}}\right)}^{0.596}$$

in which *p_a_* is the atmospheric pressure equal to 101 kPa.

**Figure 8:**
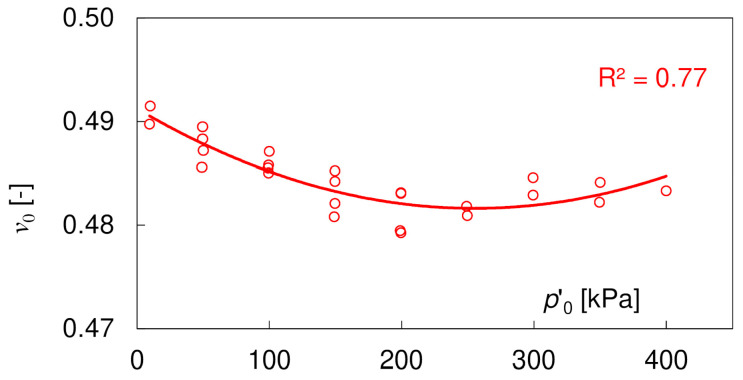
Evolution of the Poisson’s ratio *ν*_0_ (Equation (9)) with the mean effective stress *p’*_0_.

According to Equation (8), to evaluate the initial elastic modulus *E*_0_, it is necessary to know the value of the Poisson’s ratio *ν*_0_. The values obtained from the PETs, based on Equation (9), are presented in Figure 8, showing no significant dependence on the mean effective stress. The average *v* value is equal to 0.485 ± 0.03, indicating nearly undrained conditions during PE testing. In fully saturated specimens, the first observed P-wave belongs to water travelling with the velocity *V_P_* ≈ 1485 m/s. The measured P-wave velocities in the loess–sand mixtures increased from ca. 950 m/s at *p’*_0_ = 10 kPa to ca. 1980 m/s at *p’*_0_ = 400 kPa. They reached 1485 m/s at *p*’_0_ = 100 kPa. The *V_P_* values lower than 1485 m/s at the smallest confining stresses may result from a weaker contact between the piezoceramic element and soil. The values of the Poisson’s ratio obtained in the PE tests were much higher than in the UC tests, which can be explained with the much higher degree of saturation *S*. The increase in the value of the Poisson’s ratio with *S* is known from the literature [50,59].

The *E*_0_ values calculated with Equation (8) are shown in Figure 7b (after [21]). They can be roughly estimated as being equal to 2.97 *G*_0_. The dependence between the Young’s modulus and *p*’_0_ may be described with the following Equation (11):

(11) $$\frac{E_{0}}{p_{a}}=4085\cdot {\left(\frac{{p’}_{0}}{p_{a}}\right)}^{0.585}$$

#### 5.3.2. Shear Modulus G

**Figure 9:**
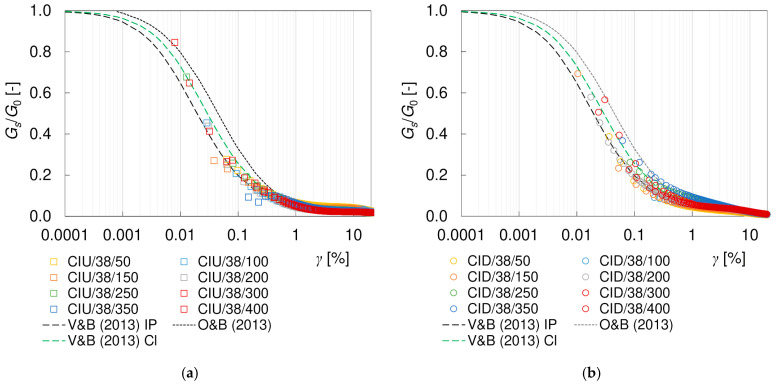
Degradation curves of the normalized secant shear modulus *G_s_*/*G*_0_ from (**a**) undrained CIU/38 and (**b**) drained CID/38 triaxial tests.

**Figure 10:**
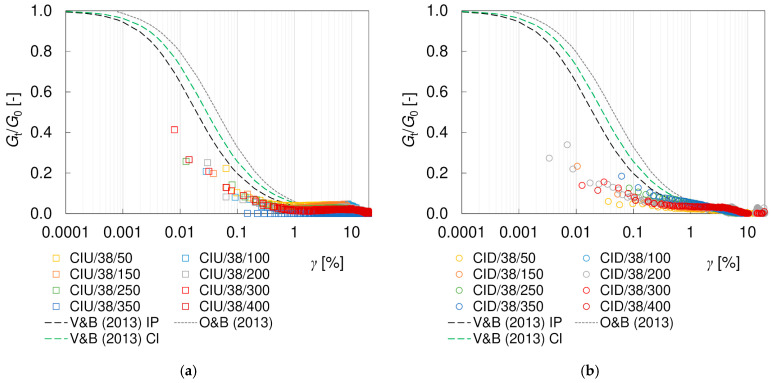
Degradation curves of the normalized tangent shear modulus *G_t_*/*G*_0_ from (**a**) undrained CIU/38 and (**b**) drained CID/38 triaxial tests.

It is well known that the shear stiffness of soil decreases with shear strain *γ*. Such characteristics were also obtained for the loess–sand mixture tested. The degradation curves of the secant and tangent shear moduli, *G_s_* and *G_t_*, are presented in Figure 9 and Figure 10. For clarity, only the CIU/38 and CID/38 specimens are included in these graphs. To eliminate the influence of the mean effective stress, the results have been normalized with *G*_0_, as calculated from Equation (10). As can be seen, the applied procedure was very effective in reducing the scatter of data, at least for the secant modulus.

There are many theoretical and empirical approaches to analytically describe the non-linear stress–strain behavior of soils [60]. The *G*/*G*_0_ degradation curve is most often modelled with a hyperbole, as first proposed by Hardin and Drnevich [61]. One of the most commonly used modifications of that proposal has been given by Darendeli [62], as follows:

(12) $$\frac{G}{G_{0}}=\frac{1}{{\left(1+\frac{\gamma }{\gamma_{ref}}\right)}^{\alpha }}$$

where *γ_ref_* is the reference shear strain and *α* is the curvature coefficient. Empirical equations and some further extensions based on this formula can be found, e.g., in [63,64,65]. For fine soils, researchers often include the plasticity index *I_P_* as a variable in the equation. This type of *G_s_*/*G*_0_ model (Equation (12)) was verified by Vardanega and Bolton [66] in a large set of the literature results for clays and silts. In static applications, they achieved the best fit at *α* = 0.736 and *γ_ref_* = 0.0022∙*I_P_* (where *I_P_* is expressed numerically, not as a percentage). In this study, the same approach with *I_P_* = 0.0564 was applied, and the results are presented in Figure 9 and Figure 10 as black dashed lines ‘V&B (2013) IP’. In terms of shape, the empirical equation shows quite a good agreement with the results of both drained and undrained secant degradation curves, although quantitatively it shall rather be treated as a lower boundary of the *G_s_*/*G*_0_ values. This non-compliance may result from very low colloidal activity (typical for loess). For fine textbook soils, the colloidal activity is around 1, meaning that the plasticity index is close to their clay content. For loess, *I_P_* is significantly higher than the content of the clay fraction and, thus, the plasticity index may be less descriptive of the soil type. In the analyzed case, the use of clay content instead of the plasticity index in the *γ_ref_* calculation gave a better fit for the degradation curves, which is shown with the green dashed lines ‘V&B (2013) Cl’. The results obtained in this study were also compared with the model for sands presented by Oztoprak and Bolton [64], in which an additional parameter *γ_e_* was introduced, shifting the degradation curve rightwards in order to describe an extended zone of elastic behavior, as follows:

(13) $$\frac{G}{G_{0}}=\frac{1}{{\left(1+\frac{\gamma -\gamma_{e}}{\gamma_{ref}}\right)}^{\alpha }}$$

If *γ_e_* = 0 is assumed, then Equation (13) reduces to Equation (12). Equation (13), with the parameters representing an average sand (*γ_ref_* = 0.044%, *γ_e_* = 0.0007%, and *α* = 0.88) was used to plot the dotted lines ‘O&B (2013)’ in Figure 9 and Figure 10. The model of Oztoprak and Bolton [64] may be considered an upper boundary for the secant moduli results obtained. Other empirical models available in the literature gave much weaker fitting to the laboratory data, and so are not discussed here.

**Figure 11:**
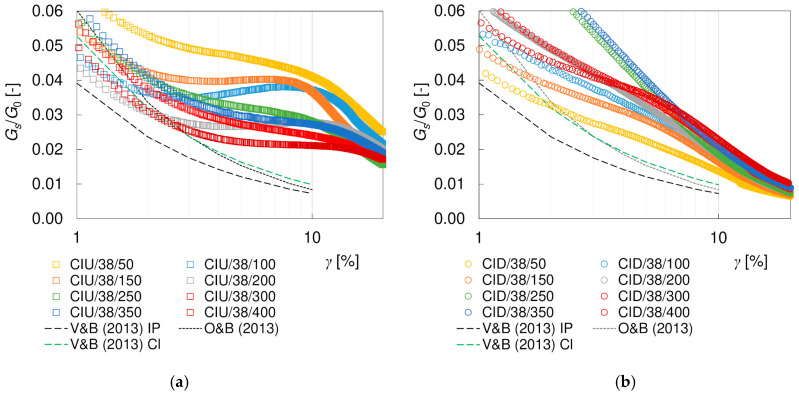
Degradation curves of the normalized secant *G_s_*/*G*_0_ shear modulus from drained CID/38 (**a**) and undrained CIU/38 (**b**) tests.

The hyperbolic models describe the course of degradation curve quite well in the small strain region. However, they are not sufficient to fully characterize the complex behavior of soils in the full range of strains. The discrepancy between the theoretical curves and the readings increases at shear strains greater than 1%, as presented in Figure 11. The influence of the drainage conditions becomes clear as well. In undrained tests, between shear strains of approximately 3–11% (which correspond to approximately 2–8% of the axial strain), a flattening of the degradation curves is visible. In drained tests, this effect is not apparent, and the stiffness degradation continues with increasing strain. Nevertheless, irrespective of the drainage conditions, the *G_s_*/*G*_0_ values at large shear strains are much higher than the theoretical predictions. However, it should be noted that the theoretical models invoked were based on different types of tests (resonant column, triaxial test, torsional shear and direct shear) and procedures with different strain range and the influence of drainage conditions was not explored in detail. The shear strain range at which the curve levelling is observed in CIU tests is not directly related to the occurrence of the (*q*)_max_ or (*q*/*p*)_max_ failure conditions. However, it seems to be connected with the cross-coupling between volumetric and distortional effects, cf. [30]. In the CID tests, the specimen initially becomes stiffer as it contracts and softens at larger strains, i.e., as it dilates, while in the undrained test, no volume changes are allowed. Due to the fact that researchers rarely perform simultaneous tests with and without drainage, it is difficult to support these considerations with the results of other studies. The explanation of this phenomenon requires further investigation.

**Figure 12:**
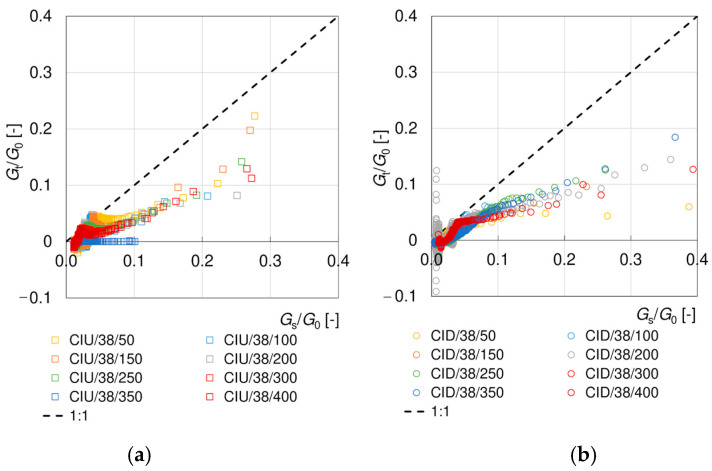
Comparison of normalized secant *G_s_*/*G*_0_ and tangent *G_t_*/*G*_0_ shear modulus from undrained CIU/38 (**a**) and undrained CID/38 (**b**) tests.

The tangent moduli obtained experimentally are smaller than the secant moduli (cf. Figure 9 and Figure 10), as expected [67]. Up to *γ*~1%, the degradation curves *G_t_/G*_0_ are located below the *G_s_/G*_0_ curves and also below the empirical models that were examined. When strain softening is observed, the tangent moduli become negative, although the absolute values of *G_t_/G*_0_ are very small. Like for the secant moduli, at shear strains greater than about 1%, the behavior depends on the drainage conditions. The difference between the values of the tangent and secant moduli obviously results from the different definitions and calculation methods. The tangent modulus is very sensitive to the smallest changes in the readings—this is why a larger scatter of values occurs for *G_t_/G*_0_, when compared to *G_s_/G*_0_. Additionally, the value of the tangent modulus depends on the range of data taken into consideration (the ^i−1^ and ^i+1^ readings) and, thus, on the frequency of the readings. There is no unambiguous relationship between the tangent and secant moduli, as presented in Figure 12. The mentioned problems are the most probable reasons why in the available literature there are much fewer data for secant moduli than for tangent moduli, and no empirical models are available to describe this degradation.

**Figure 13:**
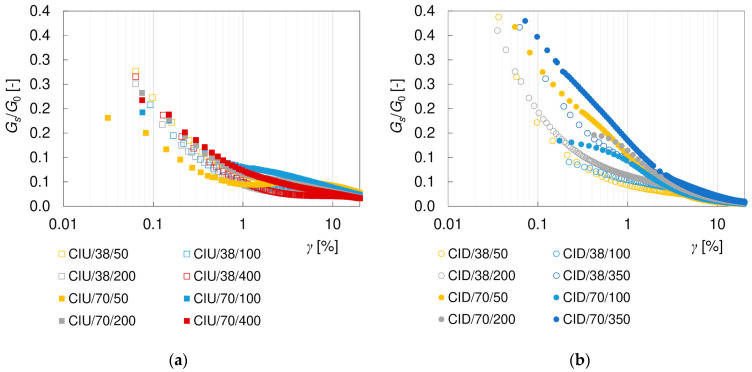
Comparison of the degradation curves *G_s_*/*G*_0_ for specimens with different sizes (38 and 70 mm in diameter) in undrained CIU (**a**) and drained CID (**b**) triaxial tests.

It was found that in the drained tests, at the shear strains of up to 3–4%, the *G_s_*/*G*_0_ values were generally larger (slower stiffness degradation) in the 70 mm diameter specimens than in the smaller ones, which is visible in Figure 13b. This is consistent with the observations from the UC tests, cf. the *E*_50_ values in Table 4. It can be noticed that the curves CID/70/100 and CID/70/200 are of different shapes than the curves CID/70/50 and CID/70/350, which results from the fact that in these tests the readings of the local displacement sensors were not available. In the CID/38 tests, the differences between the external and local strain readings were much smaller, and no difference in the shape of the curve was found. In the undrained tests (Figure 13a), up to 10% shear strain, the moduli of the larger specimens were also higher than the ones of smaller specimen. No flattening effect was observed in the 70 mm-diameter specimens. However, the CIU/70/50 specimen was exceptional. As the stiffness calculated on the basis of external sensors is usually smaller than that obtained using local ones, the shear moduli in the CIU/70 series would probably be even higher, but the trend is comparable. The number of specimens examined is too small to draw general conclusions, but the data presented indicate that the specimen size can have a significant effect on the values of the shear moduli and their strain degradation. Furthermore, evaluation of the degradation curves based on the external displacement measurements shall be avoided due to the possible seating and bedding errors, as mentioned earlier.

**Figure 14:**
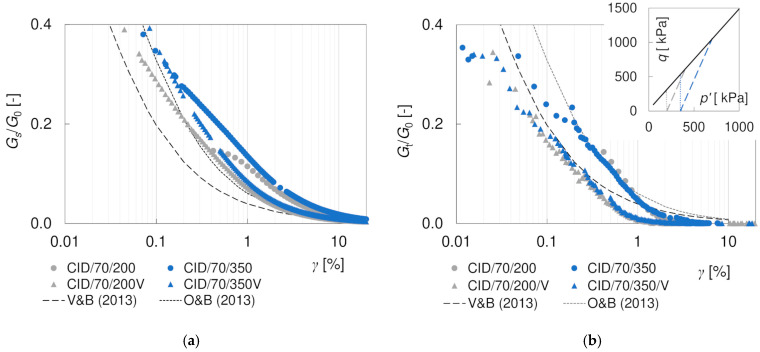
Comparison of the degradation curves *G_s_*/*G*_0_ (**a**) and *G_t_*/*G*_0_ (**b**) for specimens sheared at *σ’*_3_ = const (conventional stress path) and at *p’* = const (vertical stress path).

A significant difference was observed between the stiffness degradation curves for the specimens sheared along conventional drained (*σ’_3_* = *σ’_C_* = const) and vertical (*p’* = *σ’_C_* = const) stress paths. The results are presented in Figure 14 with circles and triangles, respectively. It is worth noting that the *G*_0_ values used for normalization (207.9 MPa for *σ’_C_* = 200 kPa and 290.1 MPa for *σ’_C_* = 350 kPa) were obtained at the isotropic state of stress, corresponding to the beginning of the paths. Both the secant and the tangent moduli obtained for vertical stress paths are smaller than the moduli from conventional compression tests. This can be explained by the fact that at the same *q* the ratio *q*/*q_max_* is greater in the tests with the vertical stress path, due to the much smaller values of *q_max_* achieved. Therefore, a greater shear strain develops, and the stiffness modulus is smaller.

#### 5.3.3. Poisson’s Ratio ν and Young’s Moduli E and E_50_

**Figure 15:**
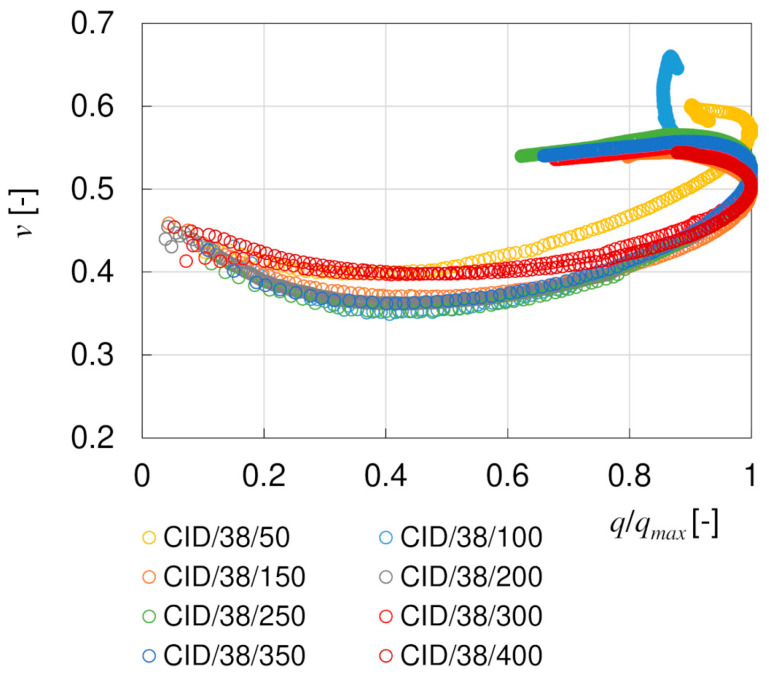
Evolution of the Poisson’s ratio *ν* with stress ratio *SF* = *q*/(*q)_max_*—CID/38 specimens.

The Poisson’s ratio *ν* is often assumed to be constant. It is obviously true in undrained triaxial tests, but not in the tests with drainage allowed. This can be seen in Figure 15, where the *ν* values of CID/38 specimens, calculated from Equation (3), are plotted in relation to the stress ratio *SF* = *q*/(*q)_max_*. The Poisson’s ratios are initially close to the *ν*_0_ values from the BE tests and gradually decrease to 0.35–0.40 as the stress intensity increases. However, starting from *SF* = 0.5, *ν* increases again, reaching values between 0.5–0.6 after the peak shear stress. A Poisson’s ratio greater than 0.5 is treated as a nonphysical value, which means that the volume of the specimen increases during shearing. Actually, this is true for strongly dilatant soils, such as the well compacted loess–sand mixture under investigation.

**Figure 16:**
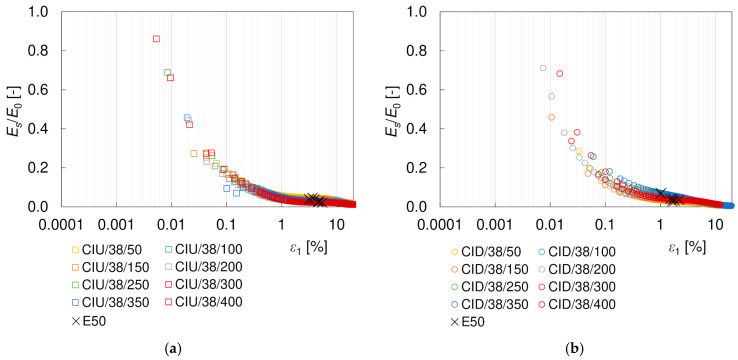
Degradation curves of the normalized secant Young’s modulus *E_s_*/*E*_0_ from undrained CIU/38 (**a**) and drained CID/38 (**b**) triaxial tests.

**Figure 17:**
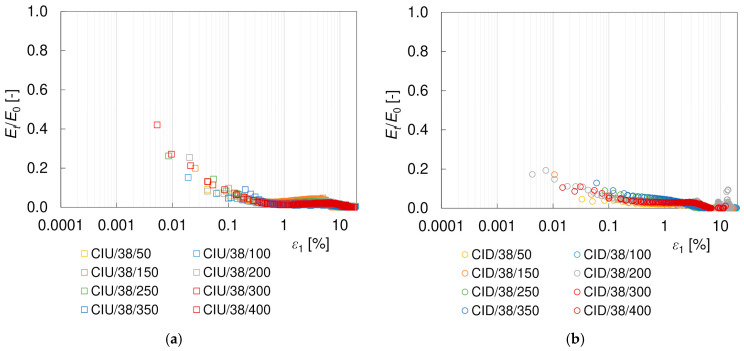
Degradation curves of the normalized tangent Young’s modulus *E_t_*/*E*_0_ from undrained CIU/38 (**a**) and drained CID/38 (**b**) triaxial tests.

In general, it is assumed that the degradation behavior of the Young’s modulus is the same as that of the shear modulus [68]. This holds true only if the Poisson’s ratio does not vary significantly during the loading process. This is not the case for the drained tests, as shown above. In the literature, Young’s modulus *E* is more often normalized with *p’*_0_ [67,69] than with *E*_0_ [34,70] or plotted against the stress ratio *SF* [68,71]. This is probably due to the difficulty in determining the initial modulus *E*_0_ in triaxial tests, resulting from the fact that the measurement of the P-wave velocity is much less available and is more troublesome than the S-wave. In this study, the values of the Young’s modulus were normalized with *E*_0_, calculated from Equation (8), to maintain a methodology similar to that used for the shear modulus. The degradation curves of the secant and tangent elastic moduli with axial strain *ε*_1_ for 38 mm diameter specimens are presented in Figure 16 and Figure 17, respectively. Some theoretical relations describing the *E/E*_0_ degradation curves can be found in [34,70,72]. However, there are practically no results for loess available in the literature to compare. Like for *G*, the tangent Young’s modulus is smaller than the shear modulus. The effect of the specimen size was also observed, as well as the influence of drainage conditions on the degradation curve at strains greater than 1%.

**Figure 18:**
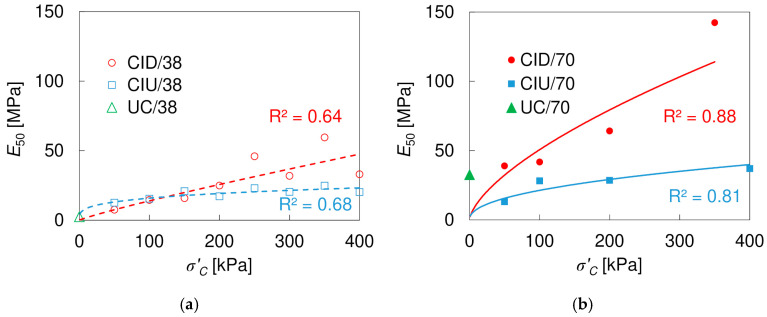
Influence of the consolidation pressure *σ’_C_* on *E*_50_ values for specimens 38 mm (**a**) and 70 mm (**b**) in diameter.

As mentioned at the beginning, *E*_50_ is the specific secant modulus that is often required in practical applications and in numerical modelling. The *E*_50_ values, together with the Poisson’s ratios *v*_50_, obtained at the same strain as *E*_50_, are shown in Table 7. The moduli and Poisson’s ratios obtained in drained and undrained tests are denoted as *E’*_50_, *v’*_50_, *E_u_*_50_, and *v_u_*_50_, respectively. The relation between *E*_50_ and consolidation pressure *σ’_C_* is also presented in Figure 18. For comparison, the values obtained in UC tests are shown at *σ’_C_* = 0 kPa, although the true effective confining stress (suction) is unknown. The relation can be described using a power function, with a reasonably good fit. It is evident that the larger specimens showed higher *E*_50_ values, both in tests with and without drainage, which is consistent with the results of the UC tests. Greater *E*_50_ values were generally obtained in the drained tests, which is related to the lower axial strains at which the stress intensity was equal to 50% of (*q)_max_*; this is visible in Figure 17, where *E*_50_ values have been marked with ‘x’ on the CID/38 and CIU/38 degradation curves. Only for the 38 mm in diameter specimens, at *σ’_C_* ≤ 150 kPa, were the *E*_50_ values slightly higher in the undrained than in the drained conditions. The results obtained in CIU tests are similar to those reported by Zarei et al. [73] for specimens of a compacted loess, 50 mm in diameter and 100 mm height, with very similar density. Contrary to the *E*_50_ modulus, which for the loess–sand mixture depends on both specimen size and consolidation pressure, the Poisson’s ratio *ν*_50_ can be assumed as constant and equal to 0.5 and 0.38 (±0.03) in the undrained and drained conditions, respectively.

## 6. Summary and Conclusions

The parameters commonly used to describe the shear strength and stiffness of soils vary depending on the testing conditions. In this study, a compacted loess with 20% sand admixture was investigated in monotonic triaxial and unconfined compression tests. Its shear strength was evaluated using different criteria. The values of shear and elastic moduli and the Poisson’s ratio have been determined, together with their dependence on strain and stress range, draining conditions, and specimen size. Both tangent and secant values of the stiffness moduli were calculated. The main conclusions are as follows:

- The *UCS* values of the loess–sand mixture were not much influenced by the size of the specimen. They were smaller than reported by other researchers for naturally compacted loess, which is due to the higher sand content and, possibly, the lower CaCO_3_ content.
- The choice of the shear strength criterion (*(q)_max_* or *(q/p)_max_*), the specimen size and drainage conditions had little influence on the value of effective angle of internal friction *φ’*, which was slightly larger than that of typical natural loess. On the other hand, the values of cohesion *c*’ depended on both the criterion used and the size of the specimens.
- The initial moduli *G*_0_ and *E*_0_ increase with consolidation pressure, and the dependency can be described with a classical power function. The values obtained for the loess–sand mixture are higher than those published for natural loess, due to the higher density and sand content, confirming the positive influence of compaction and sand admixture on loess stiffness.
- Tangent stiffness moduli show higher variability, as they are very sensitive to any changes in the slope of the stress–strain curve. Their values are smaller than the secant moduli, and after achieving maximum stress intensity, they fall below zero. There is no obvious relationship between the secant and tangent moduli.
- The larger specimens have usually been found to be stiffer. The shape of the stress path also influences the soil stiffness, which is related to the current stress level. The specimens sheared along vertical effective *p’*–*q* stress paths show faster degradation of stiffness with strain.
- The clay content is more representative for loess than *I_P_* for the theoretical description of stiffness degradation.
- The Poisson’s ratio *ν* depends on the degree of saturation and is not constant during drained shearing. No dependency on the size of the specimen was identified.
- The Young’s modulus *E* shows behavior similar to that of the shear modulus. The *E*_50_ values are greater in larger specimens, regardless of the confining stress. They are also greater in drained than in undrained triaxial tests, which is mainly due to the much smaller strain at which 50% ultimate stress intensity is achieved. Due to the high sensitivity of *E*_50_ to the testing conditions, it is not recommended as a valuable parameter in numerical analyses.

The presented study emphasizes the importance of the proper choice of testing conditions in laboratory experiments. If the pre-failure behavior is to be modelled, a number of factors must be taken into account to simulate the soil behavior in field as accurately as possible, including the range of stress and strain, loading velocity (and, thus, the drainage conditions), and the appropriate size of the specimen. It is also necessary to properly understand the definitions of the parameters required in the constitutive modelling to adequately interpret the data.

## Figures and Tables

**Table 1:** Physical parameters of the loess–sand mixture.

Sa [%]	Si [%]	Cl [%]	*d*_50_ [mm]	*C*_U_ [-]	*C*_C_ [-]	*w*_P_ [%]	*w*_L_ [%]	*I*_P_ [%]	*A* [%]	*LOI* [%]	*ρ*_s_ [g/cm^3^]	*ρ*_d.max_ [g/cm^3^]	*w*_opt_ [%]
22.9	68.6	8.5	0.36	15.0	3.4	19.9	25.5	5.64	0.66	1.9	2.66	1.86	11.0

**Table 2:** Summary of specimens for unconfined compression tests.

Symbol	Diameter [mm]	*ρ*_0_ [g/cm^3^]	*e*_0_ [-]
UC/38	38	2.069 ± 0.016	0.428 ± 0.011
UC/70	70	2.084 ± 0.006	0.417 ± 0.004

**Table 3:** Summary of specimens for triaxial compression tests.

No.	Symbol	Stress Path	Diameter [mm]	Drainage	LD	PETs	*σ’*_C_ [kPa]	*ρ*_0_ [g/cm^3^]	*e*_0_ [-]
1	CIU/38/50	Conventional	38	U	LVDT	NO	50	2.041	0.447
2	CIU/38/100	Conventional	38	U	LVDT	NO	100	2.044	0.445
3	CIU/38/150	Conventional	38	U	LVDT	NO	150	2.079	0.420
4	CIU/38/200	Conventional	38	U	LVDT	NO	200	2.056	0.430
5	CIU/38/250	Conventional	38	U	LVDT	NO	250	2.068	0.428
6	CIU/38/300	Conventional	38	U	LVDT	NO	300	2.084	0.417
7	CIU/38/350	Conventional	38	U	-	NO	350	2.103	0.404
8	CIU/38/400	Conventional	38	U	LVDT	NO	400	2.064	0.430
9	CIU/70/50	Conventional	70	U	-	NO	50	2.090	0.413
10	CIU/70/100	Conventional	70	U	-	NO	100	2.064	0.430
11	CIU/70/200	Conventional	70	U	-	NO	200	2.057	0.436
12	CIU/70/400	Conventional	70	U	-	NO	400	2.081	0.419
13	CID/38/50	Conventional	38	D	LVDT	NO	50	2.051	0.439
14	CID/38/100	Conventional	38	D	LVDT	NO	100	2.046	0.443
15	CID/38/150	Conventional	38	D	LVDT	NO	150	2.076	0.422
16	CID/38/200	Conventional	38	D	LVDT	NO	200	2.105	0.403
17	CID/38/250	Conventional	38	D	LVDT	NO	250	2.058	0.435
18	CID/38/300	Conventional	38	D	LVDT	NO	300	2.072	0.425
19	CID/38/350	Conventional	38	D	-	NO	350	2.085	0.416
20	CID/38/400	Conventional	38	D	LVDT	NO	400	2.086	0.415
21	CID/70/50	Conventional	70	D	Hall-effect	YES	50	2.027	0.456
22	CID/70/100	Conventional	70	D	-	YES	100	2.034	0.452
23	CID/70/200	Conventional	70	D	-	YES	200	2.054	0.438
24	CID/70/350	Conventional	70	D	Hall-effect	YES	350	2.070	0.427
25	CID/70/200/V	Vertical	70	D	Hall-effect	YES	200	2.022	0.456
26	CID/70/350/V	Vertical	70	D	Hall-effect	YES	350	2.032	0.448

Legend: PETs = piezoceramic element tests; *σ’*_C_ = consolidation pressure; U = undrained; D = drained; LD = type of local displacement sensors.

**Table 4:** Average strength and stiffness parameters from unconfined compression tests.

Specimen Diameter	*UCS* [kPa]		*E*_50_ [MPa]		*ν*_50_ [-]
	*ex*	*loc*	*ex*	*loc*	*loc*
38 mm	131.3 ± 11.3	137.6 ± 12.5	2.0 ± 0.4	2.4 ± 0.7	0.13 ± 0.02
70 mm	116.5 ± 4.8	118.0 ± 4.9	7.4 ± 1.5	32.5 ± 8.9	0.16 ± 0.03 *

*loc*—based on the readings of local on-specimen sensors; *ex*—based on the readings of the external sensor; * results of only 4 specimens are considered.

**Table 5:** Shear strength parameters from triaxial compression tests.

Criterion	(*q*/*p’*)_max_		(*q*)_max_	
	*φ’* [°]	*c’* [kPa]	*φ’* [°]	*c’* [kPa]
CIU/38	36.2	16.1	36.0	0.0 *
CIU/70	38.0	10.0	34.6	0.0 *
CID/38	38.5	22.8	38.5	22.7
CID/70	36.1	10.1	36.1	10.0

* Forced zero.

**Table 6:** Peak shear strength parameters of natural loess compacted at *w* ≈ *w_opt_* from other studies.

Ref.	Method ^a^	Source	Sa [%]	Cl [%]	*w_P_* [%]	*w_L_* [%]	*w_opt_* [%]	*ρ_d_* [kPa]	crit. ^b^	*φ’* [°]	*c’* [kPa]
[11]	DS/63.5	Southwestern Indiana loess (USA)	10	20	9.3	32.2	16.2	1.71	P	33.4	33.2
[56]	CID/50	Córdoba (Argentina)	0	n.d.	21.1	24.5	43.2 ^c^	1.24	6%/Y	24/6	0/3
[57]	CID/50	Khon Kaen (Thailand)	53	20	12	13	8.5 ^d^	1.95	20%	31	44
[16]	DS/150	Beshahr (Iran)	20	25	21	26	15	1.71	P	26.1	39.0
	CID/52								P	23.6	17.9
[15]	CIU/39	Shaanxi Province (China)	12	6	n.d.	n.d.	15.0 ^c^	1.4–1.7	P	28.5–38	2–5
[12]	CIU/50	Gansu Province (China)	16	6	19.4	28.6	17.2 ^e^	1.65	P	38.5	25.3

^a^ DS/x = direct shear test on specimens x mm in width or diameter, CID/x = consolidated/drained triaxial tests on saturated specimens x mm in diameter; ^b^ criterion: P = peak shear strength ((*q*)_max_/(*τ*)_max_), x% = at x% axial strain, Y = at yielding; ^c^ initial moisture content (*w_opt_* unknown); ^d^ *w* = 6.5%; ^e^ specimens compacted at *w* = 5% and *ρ_d_*/*ρ_d.max_* = 95% and then vacuum saturated; n.d. = no data.

**Table 7:** Secant Young’s modulus *E*_50_ and the corresponding Poisson’s ratio *ν*_50_.

Specimen Size	38 mm				70 mm			
*σ’*_C_ [kPa]	*E’*_50_ [MPa]	*ν’*_50_ [-]	*E_u_*_50_ [MPa]	*ν_u_*_50_ [-]	*E’*_50_ [MPa]	*ν’*_50_ [-]	*E_u_*_50_ [MPa]	*ν_u_*_50_ [-]
50	7.5	0.41	12.5	0.5	39.0	0.31	13.1	0.5
100	14.5	0.36	15.4		41.7	0.41	28.2	
150	15.8	0.37	20.8		-	-	-	
200	24.8	0.36	17.2		64.1	0.41	28.5	
250	45.9	0.35	23.0		-	-	-	
300	31.9	0.40	20.4		-	-	-	
350	59.5	0.37	24.8		142.3	0.38	-	
400	33.0	0.40	20.2		-	-	36.9	

## Data Availability

The original contributions presented in the study are included in the article, and further inquiries can be directed to the corresponding author.
